# Effects of retrograde autologous priming in adult patients undergoing cardiac surgery

**DOI:** 10.1186/1749-8090-8-S1-P66

**Published:** 2013-09-11

**Authors:** B Hofmann, A Petrov, A Abduli, M Stiller, J Naumann, T Neitzel, B Ludwig-Kraus, R-E Silber

**Affiliations:** 1 Dep. of Cardiothoracic Surgery, University Hospital, Halle (Saale), Germany; 2 Dep. of Laboratory Medicine, University Hospital, Halle (Saale), Germany

## Background

Adult cardiac surgery with extracorporeal circulation (ECC) is known to be associated with increased risk of blood transfusion and systemic inflammatory response leading to adverse outcomes. Modern procedures like retrograde autologous priming (RAP) may reduce these negative side effects of ECC. This randomized prospective study was initiated to assess whether RAP using specifically designed RAP bag (Terumo) has immediate effects on patient outcome.

## Methods

Fifty adults undergoing elective CABG or elective aortic valve replacement were randomly assigned by a computer program into two groups: the RAP group (n=25) in which the retrograde autologous priming was applied and the non-RAP (n=25) group in which the same setting was used but without the possibility to save priming volume in RAP bag. Patient demographics, preoperative characteristics and postoperative outcomes were analyzed for both groups.

## Results

There were no significant differences in operation time, blood loss and transfusion rates. No deaths and no myocardial infarctions were observed. However, RAP managed patients had a significantly lower platelet decline (p=0.004) after ECC, less catecholaminergic support (p=0.04) and a shorter intensive care unit stay (p=0.05). RAP could reduce the priming volume of the ECC up to 490 ml.

## Conclusion

Retrograde autologous priming is a safe and less invasive procedure which achieves clear benefits for adult cardiac surgery patients. Larger, sufficiently powered, study is needed to assess full benefit of this approach.

